# Prevalence and changes in boredom, anxiety and well-being among Ghanaians during the COVID-19 pandemic: a population-based study

**DOI:** 10.1186/s12889-021-10998-0

**Published:** 2021-05-26

**Authors:** Godfred O. Boateng, David Teye Doku, Nancy Innocentia Ebu Enyan, Samuel Asiedu Owusu, Irene Korkoi Aboh, Ruby Victoria Kodom, Benard Ekumah, Reginald Quansah, Sheila A. Boamah, Dorcas Obiri-Yeboah, Epaphrodite Nsabimana, Stefan Jansen, Frederick Ato Armah

**Affiliations:** 1 Department of Kinesiology, College of Nursing and Health Innovations, The University of Texas at Arlington, Arlington, TX USA; 2 Department of Population and Health, College of Humanities and Legal Studies, University of Cape Coast, Cape Coast, Ghana; 3 Directorate of Research, Innovation and Consultancy, University of Cape Coast, Cape Coast, Ghana; 4 School of Nursing and Midwifery, University of Cape Coast, Cape Coast, Ghana; 5 Department of Distance Education, University of Ghana, Accra, Ghana; 6 Department of Environmental Science, University of Cape Coast, Cape Coast, Ghana; 7 Department of Biological and Environmental Health Sciences, School of Public Health, University of Ghana, Accra, Ghana; 8 School of Nursing, McMaster University, Hamilton, Canada; 9 Department of Microbiology and Immunology, School of Medical Sciences, University of Cape Coast, Cape Coast, Ghana; 10 Mental Health & Behavior Research Group, College of Medicine and Health Sciences, University of Rwanda, Kigali, Rwanda

**Keywords:** Ghana, Psychological well-being, Coronavirus, Infectious disease, Mental health

## Abstract

**Background:**

The outbreak of the COVID-19 pandemic has been associated with several adverse health outcomes. However, few studies in sub-Saharan Africa have examined its deleterious consequences on mental health. Therefore, we investigated the prevalence and changes in boredom, anxiety and psychological well-being before and during the COVID-19 pandemic in Ghana.

**Methods:**

Data for this study were drawn from an online survey of 811 participants that collected retrospective information on mental health measures including symptoms of generalized anxiety disorder, boredom, and well-being. Additional data were collected on COVID-19 related measures, biosocial (e.g. age and sex) and sociocultural factors (e.g., education, occupation, marital status). Following descriptive and psychometric evaluation of measures used, multiple linear regression was used to assess the relationships between predictor variables and boredom, anxiety and psychological well-being scores during the pandemic. Second, we assessed the effect of anxiety on psychological well-being. Next, we assessed predictors of the changes in boredom, anxiety, and well-being.

**Results:**

Before the COVID-19 pandemic, 63.5% reported better well-being, 11.6% symptoms of anxiety, and 29.6% symptoms of boredom. Comparing experiences before and during the pandemic, there was an increase in boredom and anxiety symptomatology, and a decrease in well-being mean scores. The adjusted model shows participants with existing medical conditions had higher scores on boredom (ß = 1.76, *p* < *.001*) and anxiety (ß = 1.83, *p* < *.01*). In a separate model, anxiety scores before the pandemic (ß = -0.25, *p* < .01) and having prior medical conditions (ß = -1.53, *p* < *.001*) were associated with decreased psychological well-being scores during the pandemic. In the change model, having a prior medical condition was associated with an increasing change in boredom, anxiety, and well-being. Older age was associated with decreasing changes in boredom and well-being scores.

**Conclusions:**

This study is the first in Ghana to provide evidence of the changes in boredom, anxiety and psychological well-being during the COVID-19 pandemic. The findings underscore the need for the inclusion of mental health interventions as part of the current pandemic control protocol and public health preparedness towards infectious disease outbreaks.

**Supplementary Information:**

The online version contains supplementary material available at 10.1186/s12889-021-10998-0.

## Background

During a pandemic, the fear of spread and lethality of a disease can create anxiety, stress and depression with lasting psychological impact on the overall health and well-being of the population [1]. To contain the spread of this highly contagious novel coronavirus disease (COVID-19), many countries imposed various preventive measures including social distancing, isolation measures, mandatory and self-quarantine for persons who traveled from affected countries or those suspected to have been in contact with exposed or infected persons. While these containment measures might have contributed to the protection of the public’s health, they have implications for mental health outcomes such as anxiety, stress, boredom, negative religious coping, extreme hopelessness, suicidal ideation, and the well-being of populations at the individual, household and community levels [2].

The scale of the pandemic, the fatality of the disease, the widespread misinformation about the disease, media scrutiny and constant reporting of the deaths rather than recoveries can create fear of the unknown (anxiety), feelings of emotional and physical tensions (stress) which results from the body’s response to threats [3]. These stresses and anxieties are also associated with fears about family safety and contamination, fears about economic consequences such as job losses, fears about wage loss, and an increase in compulsive checking and search for reassurance [4–6]. These psychological effects could lead to an increase in stigmatization and xenophobia [4]. Next, a pandemic of this magnitude exposes many people to the death of loved ones and friends, including the death of their children, which can be traumatizing [4–7]. Furthermore, the prolonged time in solitude due to quarantine measures could have detrimental consequences on the psychological well-being of the most vulnerable in society [8–10]. This has been associated with high stress levels, depression, irritability and insomnia, and boredom as people are limited in their desires and engagement in social activities [8–11], impacting on their overall psychological well-being.

Psychological well-being refers to the inter- and intra-individual levels of positive function that include one’s relatedness with others and self-referent attitudes such as one’s sense of mastery and personal growth [3]. This takes two forms: subjective well-being, which focuses on the hedonic principles of pleasure and happiness of the individual. The other aspect of psychological well-being is rooted in living a good life or having a good spirit. Subjective well-being is commonly assessed by life satisfaction, the presence of positive mood, and the absence of negative mood [12, 13]. Living a good life or having a good spirit is measured in terms of autonomy, personal growth, self-acceptance, purpose in life, environmental mastery, and positive relatedness. In both cases, a sudden life event or a crisis situation could threaten the well-being of the individual, with contemporaneous health consequences [12, 13].

The fear of getting infected with the coronavirus threatens not only psychological well-being of populations, but also their physical, intellectual, emotional, occupational and spiritual well-being [14]. The effect of the disease on the psychological well-being can vary geographically and across population groups. In low-and-middle income countries where the healthcare delivery system is generally inadequate and emergency health care may be limited, the presence of a pandemic such as the COVID-19 poses a real threat [15], which may affect mental health and well-being [16]. To date, there is a dearth of research on the effect of the pandemic on the psychological well-being of populations living in low- and middle-income countries although there is a burgeoning evidence of a decline in people’s living standards and overall well-being [10–17]. Emerging studies on COVID-19 have focused primarily on the factors influencing the well-being of populations in high income countries. Evidence of the effect of the COVID-19 pandemic on the mental health and well-being in low-and middle-income countries is critical for interventions, which could facilitate the prevention of the long-term impact of the pandemic in these countries. This study examined prevalence and changes in boredom, anxiety and well-being among Ghanaians before and during the outbreak of the COVID-19 pandemic.

## Methods

### Data source and participants

This study is part of a multi-country study on Personal and Family Coping with COVID-19 in the Global South. Ghana is one of the 10 countries participating in this study. An a priori power analysis was performed using G*Power 3.1. A total sample of 199 participants was required to achieve a generally accepted minimum level of power of 0.80 while detecting the smallest effect size (Cohen’s *dz* = .2). Data for the study in Ghana were collected between July 13 to September 30, 2020, using a web-based cross-sectional study, with a total sample of 811 participants. Convenience sampling was used in the selection of voluntary participants for this study. Participation was restricted to adults aged 18 years and above and resident in Ghana. Interested participants responded to our survey via different electronic media: social media (e.g., WhatsApp groups, Facebook, Twitter, and related websites), and emails. All participants automatically received a summary of their responses instantly upon completion of the questionnaire. Statistics from the COVID-19 tracker shows the number of confirmed cases at the beginning of the survey was 25, 252 with 6491 actives cases and a seven-day average of 391 new cases. This number increased to 46,626 confirmed cases with 674 active cases and a seven-day average of 63 by the end of data collection [18].

Apart from compositional data on sex, age, place of residence, parity, education, and employment status, participants responded to questions about symptoms of boredom, anxiety, and well-being before and during the COVID-19 pandemic. They also responded to questions on coping strategies adopted and challenges managing the fears around the pandemic. Particularly, data were collected using validated instruments on boredom, loneliness, parenting, positive parenting program relationship quality, anxiety disorder, and well-being [19]. All scales were adapted to ask the questions in a ‘Before COVID-19’ and ‘Since COVID-19’ format. For instance, one of the questions on participant’s well-being before COVID-19 was expressed as “In the two weeks before the COVID-19 outbreak in Ghana, I felt cheerful and in good spirits.” The question reflecting their experience during the outbreak was expressed as “Since the beginning of COVID-19 pandemic, I have felt cheerful and in good spirits.” Using this approach, we were able to assess differences in participants’ experiences of boredom, anxiety, and well-being and the factors that may have contributed to these detrimental effects. All data based on participants’ reported experiences before COVID-19 were asked retrospectively.

### Measures

#### Outcome variables

Three outcome variables were used to assess the psychological well-being of participants. The World Health Organization (WHO)-Five Well-Being Index [19], which consists of five questions was used to assess the mental well-being of participants before and since the outbreak of COVID-19, with a response scale of ‘at no time’ coded as 0 (at no time) to 5 (all the time). It is expected that this scale will yield a score ranging from 0 to 25, with 0 representing worst possible quality of life or depression and 25 representing best possible quality of life or better well-being. A cut-off score of 13 was used to determine participants who had better perception of well-being scores and those who may have symptoms of depression or poor well-being [20, 21]. The measure of internal consistency before (α = 0.86) and after (α = 0.88) were all above the recommended threshold of 0.70.

Boredom was assessed using the short form of the Boredom Proneness Scale [22]. This consisted of eight items, which asked participants questions such as “I find it hard to entertain myself”, “I don’t feel motivated by most things that I do”, and “Much of the time, I just sit around doing nothing.” These questions were asked in the context of before and since the COVID-19 outbreak. With a seven-point Likert type response scale ranging from 1 (*strongly disagree*) to 5 (*strongly agree*), we expect the scale to produce a score ranging from 1 to 56. High scores indicate a higher tendency to experience boredom, while lower scores indicate a lower tendency to experience boredom. A cut-off score of 24 was used to determine participants who had symptoms of boredom [22]. The measure of internal consistency before (α = 0.89) and after (α = 0.91) were all above the recommended threshold of 0.70.

Anxiety symptomatology was assessed using the Generalized Anxiety Disorder 7-item (GAD-7) scale [23]. This scale asked participants how often, during the last 2 weeks, they had been bothered by each of the 7 core symptoms of generalized anxiety disorder with questions that probed into their state of nervousness, whether they had trouble relaxing or were so restless that it was hard to sit still. Response options were “not at all,” “several days,” “more than half the days,” and “nearly every day,” scored as 0, 1, 2, and 3. Consequently, the GAD-7 scores range from 0 to 21, with scores ≥5, ≥ 10, and ≥ 15 representing mild, moderate, and severe anxiety symptom levels, respectively [23]. In this study we used a cut-off ≥10 to determine participants who had symptoms of generalized anxiety disorder [23]. The measure of internal consistency before (α = 0.85) and after (α = 0.88) were all above the recommended threshold of 0.70.

#### Psychometric evaluation of outcome variables

The test of the validated factor structures of outcome variables in context of the COVID-19 crisis produced unidimensional scales for well-being, generalized anxiety disorder, and boredom proneness (Table 1). Both item-test and item-total correlations were above recommended thresholds of 0.30; also, the alpha if the item was deleted were all above recommended threshold of 0.70. Finally, the confirmatory factor loadings of each of the scale items were above the recommended threshold of 0.40.

**Table 1:** Assessing the correlations, internal consistency, and unidimensionality of adapted well-being, generalized anxiety and boredom proneness scale items

	Item-test correlation	Item-total correlation	alpha if item deleted	CFA FL (STDYX)
**Adapted WHO-5 Well-being scale: Since the beginning of COVID-19 pandemic …**				
I have felt cheerful in good spirits	0.79	0.67	0.86	0.70
I have felt calm and relaxed	0.85	0.76	0.84	0.81
I have felt active and vigorous	0.81	0.70	0.86	0.80
I woke up feeling fresh and rested	0.83	0.72	0.85	0.84
My daily life has been filled with things that interest me	0.82	0.71	0.86	0.80
**Adapted Generalized Anxiety Disorder scale: since the beginning of COVID-19 pandemic I have …**				
Being feeling nervous, anxious, or on edge	0.79	0.68	0.86	0.83
Not being able to stop or control worrying	0.80	0.71	0.86	0.86
Worrying too much about different things	0.79	0.69	0.86	0.83
Trouble relaxing	0.79	0.70	0.86	0.84
Being so restless that it is hard to sit still	0.70	0.60	0.87	0.75
Becoming easily annoyed or irritable	0.69	0.59	0.87	0.73
Feeling afraid as if something awful might happen	0.77	67.00	0.86	0.80
**Adapted Boredom Proneness Scale: since the beginning of COVID-19 pandemic...**				
1. I often find myself at “loose ends,” not knowing what to do.	0.79	0.72	0.89	0.77
2. I find it hard to entertain myself	0.79	0.71	0.89	0.76
3.Many things I have to do are repetitive and monotonous.	0.69	0.58	0.90	0.65
4. It takes more stimulation to get me going than most people.	0.79	0.72	0.89	0.80
5. I don’t feel motivated by most things that I do	0.81	0.75	0.89	0.83
6. In most situations, it is hard for me to find something to do or see to keep me interested.	0.84	0.78	0.89	0.86
7. Much of the time, I just sit around doing nothing	0.77	0.68	0.90	0.76
8.Unless I am doing something exciting, even dangerous, I feel half-dead and dull.	0.76	0.68	0.90	0.77

*WHO* World Health Organization, *alpha* Cronbach’s alpha, *CFA FL* Confirmatory Factor Analysis Factor Loadings, *STDYX* Standardized values

#### Independent variables

The independent variables used in this study for purposes of adjustment and weighting included COVID_19 related questions as well as biosocial and social cultural compositional factors. The COVID-19 related questions asked participants whether they have been infected with the virus (coded as 0 = No, 1 = Not sure, 2 = Yes), whether a member of their household had been infected (coded as 0 = No, 1 = Not sure, 2 = Yes), and whether they were concerned about COVID-19 (0 = Not to somewhat concerned, 1 = moderately concerned, 2 = extremely concerned). The biosocial factors included age (coded as 0 = 18–24, 1 = 25–34, 2 = 35–44, 3 = 45–54, 4 = 55–64, 5 = 65+) and sex (coded as 0 = female, 1 = male). The sociocultural variables consisted of place of residence (coded as 0 = Rural, 1 = Urban), occupation (coded as 0 = no employment, 1 = government employed, 2 = other employment, 3 = student), level of education (coded as 0 = Below secondary education, 1 = Post-Secondary Diploma, 2 = Bachelor’s Degree, 3 = Master’s/Doctorate), marital status (0 = single, 1 = married, 2 = divorced/separated, widowed, 3 = cohabiting, multiple partners), and number of children (0 = zero, 1 = one, 2 = two, 3 = three, 4 = four, 5 = five +).

### Data analysis

Data were analyzed using psychometric evaluative, descriptive, bivariate, and multivariable analyses. For psychometric evaluation, we reassessed each of the outcome variables for their unidimensionality, internal consistency, and construct validity to ensure they were appropriate for our analysis using Mplus 8.0 (Los Angeles, CA: Muthen & Muthen). We run a confirmatory factor analysis on each outcome variable “since the COVID-19 pandemic” to test for construct validity, unidimensionality and assessed model fitness using the Comparative Fit Index (CFI), the Tucker Lewis Index (TLI), the Root Mean Error of Approximation (RMSEA), and the Standardized Root Mean Square Residual (SRMR) [24, 25]. Best practice in scale validation suggests the following thresholds are appropriate to establish a good model fit: CFI ≥ .95, TLI ≥ .95, RMSEA ≤ .08, SRMR ≤ .08 [26, 27]. The internal consistency of the scale items was assessed using item correlations and the Cronbach’s alpha (Table 1). The model fit indices following appropriate re-specification for all three outcome variables were acceptable allowing for their use in this study (Table 2).

**Table 2:** A description of model fit indices for validated well-being, generalized anxiety disorder and boredom proneness scales

Model Fit Indices	WHO Well-Being Index	Generalized Anxiety Disorder	Boredom Proneness	Criterion
Chi-Square (*df*, *p*-value)	10.9 (4, 0.03)	85.3 (13, 0)	194.9 (28, 0)	*p* ≥ 0.05
RMSEA	0.05	0.08	0.11	≤0.08
CFI	0.99	0.99	0.98	≥0.95
TLI	0.99	0.98	0.98	≥0.95
SRMR	0.01	0.02	0.02	≤0.08

*df* degrees of freedom, *RMSEA* Root Mean Square Error of Approximation, *CFI* Comparative Fit Index, *TLI* Tucker Lewis Index, *SRMR* Standardized Root Mean Square Residual

For descriptive analysis, the general distribution of all the variables included in this study were assessed using frequency and percentages for categorical variables, and means and standard deviations for continuous or count variables. Ordinary least squares was used to assess the relationship between the outcome and predictor variables for the bivariate relationships. All variables considered to be significant at *p < .20* using a stepwise selection approach were then used to run the multivariable model. We used multiple linear regression to examine the relationship between predictor variables and the three outcome variables. We regressed boredom, anxiety and well-being mean scores on the COVID-19 related, biosocial and socio-cultural factors. We then assessed changes in boredom, anxiety and well-being symptomatology scores as a function of the predictor variables. All applicable statistical tests are two-sided and level of significance was assessed at *p < 0.05.* All analyses were completed using Stata 14.0 (StataCorp, College Station, TX, USA) and Mplus 8.0 (Los Angeles, CA: Muthen & Muthen).

## Results

### Sample characteristics

A total of 811 participants responded to the survey collected from July to September 2020. The sample was equally divided, with 50.1% being males and 49.9% females. Age ranged from 18 to 65+ (Table 3). About 56.8% of the sample were married or in some form of union. The number of children ranged from 1 to 4, with a greater proportion (44.1%) having no child. The educational level of participants depicted an elite cohort, with a greater proportion having post-secondary education (23.5%), Bachelor’s (32.4%), and Master’s (29.4%). A majority of the sample were employed (84.4%) and 59.78% described themselves to be in the lower middle-income bracket. A greater proportion of the sample lived in urban areas (79.2%) while 20.8% lived in the rural areas. About 89.0% reported no infection with COVID-19 and 90.7% reported no infection with any household member. Most of the participants were moderately (14.1%) and extremely concerned (77.4%) about the spread of COVID-19. About 81.2% had no medical condition while 11% reported having a previously diagnosed medical condition.

**Table 3:** Descriptive statistics of demographic characteristics (*n* = 811)

Sample Characteristics	Percent (%)
**Sex**	
Females	49.9
Males	50.1
Age range	
18–24	6.3
25–34	46.9
35–44	30.2
45–54	11.9
55–64	3.6
65+	1.1
**Marital status**	
Single	43.2
Married	56.8
**Number of children**	
0	44.1
1	13.1
2	17.5
3	17.3
4	5.5
More than 4	2.5
**Education**	
Secondary & lower	3.2
Post-Secondary	23.5
Bachelor’s	32.4
Master’s	29.4
Doctorate	11.5
**Employment Status**	
Unemployed	15.6
Employed	84.4
**Socio-Economic Status**	
Low income	14.9
Lower middle income	59.8
High income	1.9
Higher middle income	23.4
**Place of Residence**	
Rural	20.8
Urban	79.2
**Infection with COVID-19**	
No	89.0
Not sure	8.6
Yes	2.4
**COVID-19 infection with household member**	
No	90.7
Not sure	6.4
Yes	2.9
**Concern about COVID-19**	
Not to somewhat concern	8.5
Moderately concerned	14.1
Extremely concerned	77.4
**Existing Medical Condition**	
No condition	81.2
Not sure	7.8
Have a medical condition	11.0

Based on the recommended cut-offs and comparing scores before and during the pandemic, the percentage of those who reported to have better well-being decreased from 63.5 to 38.7%. However, the percentage of participants who could be classified as having symptoms of generalized anxiety disorder increased from 11.6 to 23.1% and those classified as experiencing symptoms of boredom increased from 29.6 to 43.2% (Supplementary Table 1).

In Table 4, we examined whether there were significant differences in symptom scores of boredom, generalized anxiety disorder and participants’ well-being. Our paired Z-test, which was used to compare the significance in scores before and during the COVID-19 crisis showed a significant difference in boredom scores (*M* = 2.68, *p* < .001), generalized anxiety disorders (*M* = 2.01, *p* < .001) and well-being scores (*M* = − 3.31, *p* < .001). Thus, there was an increase in the mean scores of symptoms of boredom and generalized anxiety disorders before and during the COVID-19 pandemic; with a decrease in well-being scores comparing before and during the COVID-19 pandemic.

**Table 4:** Changes in mean outcome scores before and during COVID-19 pandemic

Outcome variables	Paired Z test (Mean-comparison test)	95% CI
	Mean diff (Std. Err.)	
Boredom before and during COVID-19	2.68 (0.07)	[2.56, 2.80]
Generalized Anxiety Disorders before and during COVID-19	2.01 (0.03)	[1.90, 2.13]
WHO-Wellbeing before and during COVID-19	-3.31 (0.01)	[−3.43, −3.19]

*Mean diff* Mean difference, *Std. Err* Standard Error, *CI* Confidence Interval, a negative (−) mean diff suggest a decrease in mean scores whiles a positive (+) mean difference suggest an increase in mean scores

### Multivariable linear regression results predicting boredom, anxiety and well-being

Table 5 is a description of the multivariable model that examined the relationships between boredom, anxiety and well-being during the COVID-19 pandemic as well as predictor variables. Males had a decreasing score on boredom than females (ß = − 1.14, *p* < .05). Those aged 55–64 and 65 + had a decreasing score on boredom than those in aged 18–24 years. On average, a unit increase in a boredom score before COVID-19 was associated with a .89 increase in boredom scores during the COVID-19 pandemic. Also, participants with a medical condition relative to those without, had a 1.76 increase in boredom scores.

**Table 5:** A multivariable linear model showing the relationships between predictor variables and boredom, anxiety and well-being during the COVID-19 pandemic

	Boredom	Anxiety	WHO Well-Being
	ß (Std. Err)	ß (Std. Err)	ß (Std. Err)
**Sex**			
Females (Ref.)			
Males	-1.14 (0.48) *	-0.97 (0.35) **	0.74 (0.40)
**Age range**			
18–24 (Ref.)			
25–34	−2.13 (1.30)	1.07 (0.84)	0.39 (0.92)
35–44	−1.23 (1.46)	1.76 (0.94)	−0.12 (1.09)
45–54	−2.77 (1.58)	−0.35 (1.05)	0.27 (1.26)
55–64	−4.32 (2.03) *	0.04 (1.29)	0.49 (1.47)
65+	−4.49 (1.81) *	−2.23 (1.33)	4.30 (1.44) **
**Marital status**			
Single (Ref.)			
Married	−0.77 (0.75)	−1.17 (0.54) *	0.87 (0.63)
**Number of children**			
0 (Ref.)			
1	0.74 (0.75)	0.45 (0.70)	−1.21 (0.78)
2	−0.79 (0.82)	0.11 (0.64)	−0.82 (0.78)
3	0.16 (.99)	0.96 (0.73)	−0.37 (0.84)
4	−0.19 (1.59)	0.34 (0.99)	−1.48 (1.04)
More than 4	0.72 (1.20)	2.20 (1.20)	−4.28 (1.35) **
**Education**			
Secondary & lower (Ref)			
Post-Secondary	−0.43 (1.38)	1.23 (0.79)	−3.59 (1.06) ***
Bachelor’s	0.27 (1.36)	0.55 (0.77)	−3.14 (1.04) **
Master’s	0.29 (1.36)	1.30 (0.81)	−3.12 (1.07) **
Doctorate	−0.67 (1.46)	0.49 (0.93)	−3.24 (0.18) *
**Employment Status**			
Unemployed (Ref.)			
Employed	−0.59 (0.86)	0.20 (0.66)	0.14 (0.69)
**Socio-Economic Status**			
Low income (Ref.)			
Lower Middle income	−0.26 (0.84)	0.22 (0.57)	0.54 (0.64)
High income	−1.39 (1.73)	0.02 (0.79)	4.92 (1.45) *
Higher Middle income	0.60 (0.96)	−0.11 (1.15)	1.36 (0.77)
**COVID-19 infection with household member**			
No (Ref.)			
Not sure	1.19 (1.05)	1.35 (0.0.79)	−0.72 (0.75)
Yes	1.41 (1.38)	−0.12 (1.15)	0.86 (0.92)
**Boredom before COVID-19**	0.89 (0.03) ***		
**Anxiety before COVID-19**		0.59 (0.04) ***	−0.25 (0.04) ***
**Concern about COVID-19**			
Not to somewhat concern (Ref)			
Moderately concerned		0.51 (0.69)	0.65 (0.86)
Extremely concerned		1.24 (0.57) *	−1.53 (0.69)
**Existing Medical Condition**			
No condition (Ref.)			
Not sure	1.78 (1.09)	0.91 (0.39)	−0.85 (0.58)
Have a medical condition	1.76 (0.80) ***	1.83 (0.65) **	−1.53 (0.58) **
Root MSE	6.52	4.81	5.42
Adjusted R-Squared	0.59	0.31	0.11

*ß* Beta Coefficient, *Std Err* Robust Standard Error, *WHO* World Health Organization, *Root MSE* the root mean squared error is the sd of the regression. The closer to zero better the fit. *p < 0.05, **p < 0.01, *** p < 0.001

Similarly, males compared to females had lower scores on anxiety symptomatology (ß = − 0.97, *p* < .01). Also, those in a union or marital relationship had a lower score on anxiety compared to those single (ß = -0.77, *p* < .05). On average, a unit increase in anxiety scores before the COVID-19 pandemic was associated with a .59 increase in anxiety scores during the pandemic. Also, those who were extremely concerned about the COVID-19 pandemic (ß = 1.24, *p* < .05) and those who previously had a medical condition (ß = 1.83, *p* < .01) had an increasing score on anxiety during the pandemic when compared to those not too concerned and who had no prior medical condition.

In the third model on well-being, those 65 years and older had higher well-being scores (ß = 4.30, *p* < .01) than those 18–24 years. Participants who had more than 4 children and had post-secondary to a doctoral degree education level had lower well-being scores compared to their reference categories (Table 5). Additionally, those in the high-income cohort (ß = 4.92, *p* < .05) relative to those in the low-income cohort had higher well-being scores. An increase in anxiety symptomatology scores before the COVID-19 crisis and having a prior medical condition were associated with lower well-being scores during the pandemic (Table 5).

### Multivariable linear regression results predicting changes in boredom, anxiety and well-being scores

Table 6 shows the relationships between changes in boredom, anxiety, and well-being before and during the COVID-19 crisis as well as predictor variables. By subtracting the individual before scores from the during scores, we developed a change score for all the variables. Higher scores on these change variables suggested an increasing change in boredom, anxiety, and well-being; while lower scores suggested a decreasing change. On average, males compared to females had a decreasing change in the difference scores, suggesting lower boredom rates among males. Also, those in ages 55–64 and 65 years + had decreasing changes in boredom scores. Boredom before the COVID-19 pandemic and those who were moderately concerned about the pandemic had a decreasing change in the boredom differential scores. However, having a medical condition resulted in an increasing change in boredom. Thus, participants with a medical condition had an increase in their levels of boredom during the pandemic relative to their state of boredom before the COVID-19 crisis.

**Table 6:** A multivariable linear model showing the relationships between changes in boredom, anxiety and well-being during COVID-19 lock down and predictor variables

	△ Boredom	△ Anxiety	△ WHO Well-Being
	ß (Std. Err)	ß (Std. Err)	ß (Std. Err)
**Sex**			
Females (Ref.)			
Males	−1.04 (0.48) *	−0.99 (0.35) **	− 0.17 (0.35)
**Age range**			
18–24 (Ref.)			
25–34	−1.99 (1.28)	1.10 (0.84)	−1.14 (0.69)
35–44	−1.14 (1.45)	1.77 (0.95)	−1.92 (0.84) *
45–54	−2.82 (1.58)	− 0.35 (1.05)	− 2.08 (0.97) *
55–64	−4.22 (2.03) *	0.07 (1.29)	−4.31 (1.37) **
65+	−4.32 (1.83) *	−2.29 (1.33)	−1.48 (1.17)
**Marital status**			
Single (Ref.)			
Married	−0.71 (0.75)	−1.17 (0.54) *	0.25 (0.51)
**Number of children**			
0 (Ref.)			
1	0.75 (0.98)	0.45 (0.71)	−0.02 (0.62)
2	−0.90 (0.84)	0.14 (0.64)	−0.69 (0.65)
3	0.13 (1.02)	0.97 (0.74)	−0.02 (0.67)
4	−0.22 (1.62)	0.33 (0.99)	0.06 (1.04)
More than 4	0.57 (1.24)	2.26 (1.20)	−2.04 (1.29)
**Education**			
Secondary & lower (Ref)			
Post-Secondary	−0.41 (1.34)	0.57 (0.79)	2.34 (0.83) ***
Bachelor’s	−0.73 (0.68)	0.66 (0.48)	−1.07 (0.50) *
Master’s	0.01 (0.69)	0.72 (0.48)	0.22 (0.45)
Doctorate	−1.00 (0.88)	−0.13 (0.68)	−0.12 (0.71)
**Employment Status**			
Unemployed (Ref.)			
Employed	−0.63 (0.86)	0.24 (0.66)	0.47 (0.57)
**Socio-Economic Status**			
Low income (Ref.)			
Lower Middle income	−1.38 (1.25)	0.22 (0.57)	0.54 (0.64)
High income	−0.53 (0.95)	0.10 (0.58)	4.92 (1.45)
Higher Middle income	0.21 (0.83)	−0.10 (0.64)	1.36 (0.77)
**Infection with COVID-19**			
No (Ref.)			
Not sure	1.38 (1.25)	−0.06 (0.93)	0.01 (0.69)
Yes	1.61 (1.42)	−1.41 (1.13)	0.03 (0.40)
**COVID-19 infection with household member**			
No (Ref.)			
Not sure	−0.29 (1.00)	−1.45 (1.03)	0.09 (0.72)
Yes	−0.10 (1.84)	0.19 (1.18)	−0.67 (0.80)
**Boredom before COVID-19**	−0.11 (0.03) ***		
**Anxiety before COVID-19**		−0.41 (0.04) ***	−0.31 (0.04) ***
**Concern about COVID-19**			
Not to somewhat concern (Ref)			
Moderately concerned	−0.29 (1.00) *	0.47 (0.69)	0.01 (0.69)
Extremely concerned	−0.09 (0.84)	1.19 (0.57) *	−0.03 (0.93)
**Existing Medical Condition**			
No condition (Ref.)			
Not sure	1.77 (1.09)	0.89 (0.63)	0.51 (0.65)
Have a medical condition	1.78 (0.81) *	1.87 (0.65) **	0.96 (0.54) **
Root MSE	6.53	4.82	4.75
Adjusted R-Squared	0.07	0.20	0.11

*Coef* Coefficient, *Std Err* Robust Standard Error, *WHO* World Health Organization, *Root MSE* the root mean squared error is the sd of the regression. The closer to zero better the fit.; △ = Change in; *p < 0.05, **p < 0.01, *** p < 0.001;

In terms of anxiety, males relative to females on average, had a 0.99 decreasing change in anxiety. Similarly, those married or in a union had a decreasing change in anxiety relative to those single. However, participants who were extremely concerned (ß = 1.19, *p* < 0.05) and those who had a prior medical condition (ß = 4.92, *p* < 0.05) had an increasing change in anxiety. Thus, their levels of anxiety increased during the pandemic compared to before COVID-19.

For well-being, increasing age cohorts was associated with decreasing changes in well-being. Thus, those who were aging had lower scores on well-being during the COVID-19 pandemic relative to its onset. However, participants with post-secondary education relative to those with secondary or lower education had an increasing change in well-being scores. On average, participants who reported having anxiety before COVID-19 experienced decreasing changes in well-being during the pandemic. Finally, having a prior medical condition appears to be associated with an increasing change in well-being scores during the pandemic (Table 6).

## Discussion

To our knowledge, this study is the first to investigate the predictors and conditions around COVID-19 pandemic on boredom, anxiety, and well-being in Ghana’s population. Our data suggest an increase in symptoms of boredom and anxiety, and a decrease in well-being during the COVID-19 pandemic compared to before COVID-19. These findings are consistent with previous studies which suggest that public levels of anxiety increase during major disease outbreaks or pandemics [28–30].

The increase in anxiety and boredom is an important finding in understanding the mental health conditions of Ghanaians during the COVID-19 pandemic. The fear of contracting the disease or dying, stigmatization against persons who contract the disease or their close acquaintances, shortage of essential supplies, the uncertainty of the pandemic and feeling helpless during the lockdown period may have created anxiety [6]. Similarly, the strict social distancing regulations, isolation and quarantine, and partial lockdowns associated with the COVID-19 protocol in Ghana might have reduced social contacts and can explain the increase in boredom. Thus, disruption in social contacts is likely to have contributed to the anxiety in the population [4–6]. Boredom can lead to loneliness and this can have indirect effect on population health; for example, engagement in health compromising behaviours such as substance use/drug abuse may have direct effects on psychological health [31].

Some earlier studies also reported increase in boredom during the COVID-19 pandemic [32, 33]. In the Ghanaian society where close social contact is pervasive in the culture [34], isolation, quarantine, and lockdown can be very traumatic to the people thereby creating negative effects on their mental health in general [35]. Boredom can lead to surfing of social network sites, which usually carry “doomsday” news, as evident with the COVID-19 pandemic [36, 37]. Boredom can also lead to poor psychological health [32].

Next, our study findings suggest a significant amplification of symptoms of psychological disorders in the Ghanaian population during the COVID-19 pandemic. Specifically, the results attest to the worsening of experiences related to boredom and anxiety during the pandemic relative to participants experiences before the pandemic. In both cases, those with existing medical conditions experienced increased forms of boredom and anxiety symptomatology. Furthermore, anxiety scores before the pandemic and having prior medical condition were found to be associated with a decline in the well-being of participants. Furthermore, people who were extremely concerned about the disease had increased anxiety whereas older people experienced a decline in boredom and an increase in well-being.

Another significant finding, related to the effect of pre-existing health conditions on the well-being of the Ghanaian population. Indeed, prior studies show that people with pre-existing comorbidities have a higher risk of severe COVID-19 infection or related deaths [38, 39]. People with underlying medical conditions might have initiated self-imposed quarantine, social and physical distancing measures long before official lockdowns were imposed. Consequently, those with a medical condition might have longer social disruptions and subsequently higher levels of boredom than those without pre-existing medical conditions. This is consistent with the findings in our study, as participants with pre-existing chronic conditions had higher boredom and anxiety scores but lower well-being scores. It is also possible because persons with pre-existing medical conditions have an increased risk of COVID-19 infection and death. Further research that investigates the mechanisms linking pre-existing chronic conditions, boredom and anxiety during the pandemic will shed light on the present findings. Additionally, boredom during a disaster is known to lead to stress and anxiety [40, 41]. Hence, it is not surprising that we found increased anxiety among people with medical conditions during the pandemic.

Surprising to the research team, participants with higher levels of education including those with post-secondary to doctoral education had lower well-being scores compared with those having secondary school or lower levels of education. Also, an increasing change in well-being during the pandemic was found among those who had post-secondary education while those who had Bachelor’s degree had a decreasing change in their well-being when compared to those with secondary or lower education. These findings are at odds with the social gradient of health theory which suggests that those with poor social status have worse health while those with a better social status would have better health outcomes. In this study, the opposite is what is noted as those with lower educational levels have better well-being than those with Bachelor’s or doctoral degrees. While we did not investigate this finding, it is possible that the poorly educated had become immune to social and economic shocks facilitating their ability to cope and respond adequately to the COVID-19 due to availability of interpersonal support or resources [42] while the well-educated, who may have experienced an abrupt change in their income and style of living had difficulty adjusting to the pandemic, leading to a decline in their well-being. This notwithstanding, these counterintuitive findings warrant further investigation.

Furthermore, our data suggested that males had lower levels of anxiety and boredom than females. This was consistent with earlier studies [42–44]. One plausible explanation is that females in this context may spend a great deal of time worrying about the pandemic because of their caring role at home which might have increased during the pandemic as schools were closed and children stayed home. Our finding however differed from a Chinese study which found no difference in anxiety disorders and mood by sex during the COVID-19 pandemic [45]. Also, married persons had a decreased change in anxiety compared to those who were single. This is consistent with the buffering hypothesis which suggests that under some conditions social support protects individuals from the harmful effects of stressful conditions [42]. In this study, our findings show that spousal support may have served as a buffer against the stress and anxiety associated with the pandemic.

Next, our study also highlights the validity and reliability of existing scales which can be adapted successfully for use in research on mental health correlates of COVID-19. In this study, we successfully adapted, tested and validated the hypothetical factor structure of the boredom proneness scale, the generalized anxiety disorder scale, and the WHO-5 well-being scale to COVID-19 conditions. These scales exemplified satisfactory model fitness and acceptable reliability thresholds, making them user friendly for studies related to infectious diseases.

Generally, our study provides important findings which can inform preparedness for future outbreak; however, there are some limitations which provide opportunities for future research. The cross-sectional nature of the study does not allow for causal inferences. Future studies may consider a longitudinal study with a randomized controlled design to establish causal effects. Indeed, the study design in subsequent research should actually assess participants health status before and during an outbreak. Second, because of the lockdown and to avoid the possible spread of the virus during data collection, the survey was web-based. Therefore, there is a possibility of selection bias which should be taken into consideration in the interpretation of the findings. However, web-based surveys are valid and reliable [46]. Also, best practice suggests the use of a longitudinal study with a randomized controlled design that actually assessed participants before and during the pandemic. However, in this study, measures on participants psychological well-being were assessed at a single timepoint and based on a retrospective assessment, which may have been biased either as a result of social desirability or forgetfulness. Finally, our study was limited to adults in their reproductive ages with the exclusion of minors. We propose that future studies should investigate the effect of the pandemic on the mental health of other populations such as school children.

## Conclusions

These findings underscore the indirect associations of the COVID-19 pandemic on the mental health and well-being of the Ghanaian population. In particular, people who had prior medical conditions were at increased risk of poor psychological well-being. This study has implications for policies and programs aimed at reducing the surge in COVID-19 infections. Principally, it highlights the need to provide psychological interventions especially for those with existing high risk COVID-19-related medical conditions, in addition to the routine precautionary measures. Public health preparedness towards infectious disease outbreaks should include mental health interventions in order to avert the long-term effects of such catastrophes on health and well-being.

## Supplementary Information

**Additional file 1: Supplementary Table 1.** Univariate statistics and alpha values of outcome measures used in assessing psychological well-being, and symptoms of anxiety and boredom.

## Data Availability

The datasets used and/or analysed during the current study are available from the corresponding author on reasonable request.

## Abbreviations

CFI: Comparative Fit Index
COVID-19: Coronavirus disease 2019
GAD: Generalised Anxiety Disorder
RMSEA: Root Mean Error of Approximation
SRMR: Standardized Root Mean Square Residual
TLI: Tucker Lewis Index

## References

[1] Hall RC, Hall RC, Chapman MJ (2008). The 1995 Kikwit Ebola outbreak: lessons hospitals and physicians can apply to future viral epidemics. Gen Hosp Psychiatry.

[2] Lee SA (2020). Coronavirus anxiety scale: a brief mental health screener for COVID-19 related anxiety. Death Stud.

[3] Burijon BN. Biological bases of clinical anxiety. New York: WW Norton & Co; 2007.

[4] Taylor S. The psychology of pandemics: preparing for the next global outbreak of infectious disease. New Castle: Cambridge Scholars Publishing; 2019.

[5] Taylor S, Landry CA, Paluszek MM, Fergus TA, McKay D, Asmundson GJ (2020). Development and initial validation of the COVID stress scales. J Anxiety Disord.

[6] Taylor S, Landry CA, Paluszek MM, Rachor GS, Asmundson GJ (2020). Worry, avoidance, and coping during the COVID-19 pandemic: a comprehensive network analysis. J Anxiety Disord.

[7] Taylor CA, Bell JM, Breiding MJ, Xu L (2017). Traumatic brain injury–related emergency department visits, hospitalizations, and deaths—United States, 2007 and 2013. MMWR Surveill Summ.

[8] Rossi R, Socci V, Talevi D, Mensi S, Niolu C, Pacitti F, et al. COVID-19 pandemic and lockdown measures impact on mental health among the general population in Italy. Front Psychiatry. 2020;11:790. 10.3389/fpsyt.2020.00790. 10.3389/fpsyt.2020.00790 PMC7426501 32848952

[9] Martarelli CS, Wolff W (2020). Too bored to bother? Boredom as a potential threat to the efficacy of pandemic containment measures. Hum Soc Sci Commun.

[10] Chirisa I, Mutambisi T, Chivenge M, Mabaso E, Matamanda AR, Ncube R (2020). The urban penalty of COVID-19 lockdowns across the globe: manifestations and lessons for Anglophone sub-Saharan Africa. Geo J.

[11] Boylan J, Seli P, Scholer AA, Danckert J (2021). Boredom in the COVID-19 pandemic: trait boredom proneness, the desire to act, and rule-breaking. Personal Individ Differ.

[12] Salgado JF, Blanco S, Moscoso S (2019). Subjective well-being and job performance: testing of a suppressor effect. J Work Organ Psychol.

[13] Diener E, Lucas RE, Oishi S (2002). Subjective well-being: the science of happiness and life satisfaction. Handb Positive Psychol.

[14] Patrick SW, Henkhaus LE, Zickafoose JS, Lovell K, Halvorson A, Loch S, et al. Well-being of parents and children during the COVID-19 pandemic: a national survey. Pediatrics. 2020;146(4):e2020016824. 10.1542/peds.2020-016824. 10.1542/peds.2020-016824 32709738

[15] Okereke M, Ukor NA, Adebisi YA, Ogunkola IO, Favour Iyagbaye E, Adiela Owhor G, et al. Impact of COVID-19 on access to healthcare in low-and middle-income countries: current evidence and future recommendations. Int J Health Plan Manag. 2021;36(1):13. 10.1002/hpm.3067 32857892

[16] Moitra M, Rahman M, Collins PY, Gohar F, Weaver M, Kinuthia J, et al. Mental health consequences for healthcare workers during the COVID-19 pandemic: a scoping review to draw lessons for LMICs. Front Psychiatry. 2021;12:22. 10.3389/fpsyt.2021.602614 PMC7873361 33584383

[17] Egger D, Miguel E, Warren SS, Shenoy A, Collins E, Karlan D, et al. Falling living standards during the COVID-19 crisis: Quantitative evidence from nine developing countries. Sci Adv. 2021;7(6):eabe0997. 10.1126/sciadv.abe0997 PMC7864564 33547077

[18] Ghana COVID-19 Tracker. 2020. https://www.bing.com/covid/local/ghana?vert=graph. Accessed 15 Apr 2021.

[19] Regional Office for Europe WHO. Use of Well-Being Measures in Primary Health Care - The DepCare Project: Health for All, Target 12; 1998. [http://www.who.dk/document/e60246.pdf].

[20] Psychiatry and Behavioral Health Learning Networks (2015). WHO (Five) Well-Being Index (WHO-5).

[21] Topp CW, Østergaard SD, Søndergaard S, Bech P (2015). The WHO-5 well-being index: a systematic review of the literature. Psychother Psychosom.

[22] Struk AA, Carriere JS, Cheyne JA, Danckert J (2017). A short boredom proneness scale: development and psychometric properties. Assessment.

[23] Löwe B, Decker O, Müller S, Brähler E, Schellberg D, Herzog W, et al. Validation and standardization of the generalized anxiety disorder screener (GAD-7) in the general population. Med Care. 2008;1:266–74. 10.1097/MLR.0b013e318160d093 18388841

[24] Bentler PM (1990). Comparative fit indexes in structural models. Psychol Bull.

[25] Bentler PM, Bonett DG (1980). Significance tests and goodness of fit in the analysis of covariance structures. Psychol Bull.

[26] Boateng GO, Neilands TB, Frongillo EA, Melgar-Quiñonez HR, Young SL (2018). Best practices for developing and validating scales for health, social, and behavioral research: a primer. Front Public Health.

[27] Hu L, Bentler PM (1999). Cutoff criteria for fit indexes in covariance structure analysis: conventional criteria versus new alternatives. Struct Equ Model Multidiscip J.

[28] Su TP, Lien TC, Yang CY, Su YL, Wang JH, Tsai SL, et al. Prevalence of psychiatric morbidity and psychological adaptation of the nurses in a structured SARS caring unit during outbreak: a prospective and periodic assessment study in Taiwan. J Psychiatr Res. 2007;41(1–2):119–30. 10.1016/j.jpsychires.2005.12.006. 10.1016/j.jpsychires.2005.12.006 PMC7094424 16460760

[29] Xiang YT, Yang Y, Li W, Zhang L, Zhang Q, Cheung T, et al. Timely mental health care for the 2019 novel coronavirus outbreak is urgently needed. Lancet Psychiatry. 2020;7(3):228–9. 10.1016/S2215-0366(20)30046-8. 10.1016/S2215-0366(20)30046-8 PMC7128153 32032543

[30] Khoshaim HB, Al-Sukayt A, Chinna K, Nurunnabi M, Sundarasen S, Kamaludin K, et al. Anxiety level of University students during COVID-19 in Saudi Arabia. Front Psychiatry. 2020;11:1397. 10.3389/fpsyt.2020.579750 PMC7759470 33362601

[31] Pels F, Kleinert J (2016). Loneliness and physical activity: a systematic review. Int Rev Sport Exerc Psychol.

[32] Brooks SK, Webster RK, Smith LE, Woodland L, Wessely S, Greenberg N, et al. The psychological impact of quarantine and how to reduce it: rapid review of the evidence. Lancet. 2020;26:912–20. 10.1016/S0140-6736(20)30460-8 PMC7158942 32112714

[33] Ying L, Luan S, Hertwig R (2020). Public emotion profiles in times of the COVID-19: A longitudinal study in the US and China.

[34] Kito M, Yuki M, Thomson R (2017). Relational mobility and close relationships: a socioecological approach to explain cross-cultural differences. Pers Relat.

[35] Prabha MR (2020). The trauma of being quarantined and its coping strategies. IOSR J Hum Soc Sci.

[36] Zhou SX (2012). Louis Leun, 0 l of journalism and communication.

[37] Aboujaoude E (2010). Problematic internet use: an overview. World Psychiatry.

[38] Guan WJ, Ni ZY, Hu Y, Liang WH, Ou CQ, He JX, et al. Clinical characteristics of coronavirus disease 2019 in China. N Engl J Med. 2020;382(18):1708–20. 10.1056/NEJMoa2002032. 10.1056/NEJMoa2002032 PMC7092819 32109013

[39] Chen N, Zhou M, Dong X, Qu J, Gong F, Han Y, et al. Epidemiological and clinical characteristics of 99 cases of 2019 novel coronavirus pneumonia in Wuhan, China: a descriptive study. Lancet. 2020;395(10223):507–13. 10.1016/S0140-6736(20)30211-7. 10.1016/S0140-6736(20)30211-7 PMC7135076 32007143

[40] Chao M, Chen X, Liu T, Yang H, Hall BJ (2020). Psychological distress and state boredom during the COVID-19 outbreak in China: the role of meaning in life and media use. Eur J Psychotraumatol.

[41] Lee FK, Zelman DC (2019). Boredom proneness as a predictor of depression, anxiety and stress: the moderating effects of dispositional mindfulness. Personal Individ Differ.

[42] Cohen S, Wills TA (1985). Stress, social support, and the buffering hypothesis. Psychol Bull.

[43] McLean CP, Anderson ER (2009). Brave men and timid women? A review of the gender differences in fear and anxiety. Clin Psychol Rev.

[44] Guo X, Meng Z, Huang G, Fan J, Zhou W, Ling W, et al. Meta-analysis of the prevalence of anxiety disorders in mainland China from 2000 to 2015. Sci Rep. 2016;6(1):1–5. 10.1038/srep28033 PMC4910078 27306280

[45] Gao W, Ping S, Liu X (2020). Gender differences in depression, anxiety, and stress among college students: a longitudinal study from China. J Affect Disord.

[46] Lassale C, Péneau S, Touvier M, Julia C, Galan P, Hercberg S, et al. Validity of web-based self-reported weight and height: results of the Nutrinet-Santé study. J Med Internet Res. 2013;15(8):e152. 10.2196/jmir.2575. 10.2196/jmir.2575 PMC3742400 23928492

