# Supplementary material for: Prevalence and changes in boredom, anxiety and well-being among Ghanaians during the COVID-19 pandemic: a population-based study
Source: BMC Public Health. 2021 May 26;21:985. doi: 10.1186/s12889-021-10998-0 (PMC8149916; doi:10.1186/s12889-021-10998-0)
Supplement: Supplementary file 1 — Additional file 1: Supplementary Table 1. Univariate statistics and alpha values of outcome measures used in assessing psychological well-being, and symptoms of anxiety and boredom. [file 12889_2021_10998_MOESM1_ESM.docx]

| **Outcome Measures** | (Means (SD)) | Percent (%) | Alpha |
| --- | --- | --- | --- |
| WHO well-being scores before COVID-19 | 15.19 (5.55) | 63.5 | 0.86 |
| WHO well-being scores during COVID-19 | 11.88 (5.71) | 38.7 | 0.88 |
| Generalized Anxiety Scores before COVID-19 | 3.85 (4.71) | 11.6 | 0.85 |
| Generalized Anxiety Scores during COVID-19 | 5.87 (5.68) | 23.1 | 0.88 |
| Boredom scores before COVID-19 | 20.42 (8.39) | 29.6 | 0.89 |
| Boredom scores during COVID-19 | 23.11 (10.13) | 43.3 | 0.91 |

**Supplementary Table 1**

Univariate statistics and alpha values of outcome measures used in assessing psychological well-being, and symptoms of anxiety and boredom
